# Supplementary material for: Splice-Junction-Based Mapping of Alternative Isoforms in the Human Proteome
Source: Cell Rep. Author manuscript; Available in PMC 2020 Jan 15. (PMC6961840; doi:10.1016/j.celrep.2019.11.026)
Supplement: 2 [file NIHMS1546469-supplement-2.zip › DF1/PXD010154/Liver/Liver_31_ALB_HECYAKVLSVVLNQLCVLHEK.pdf]

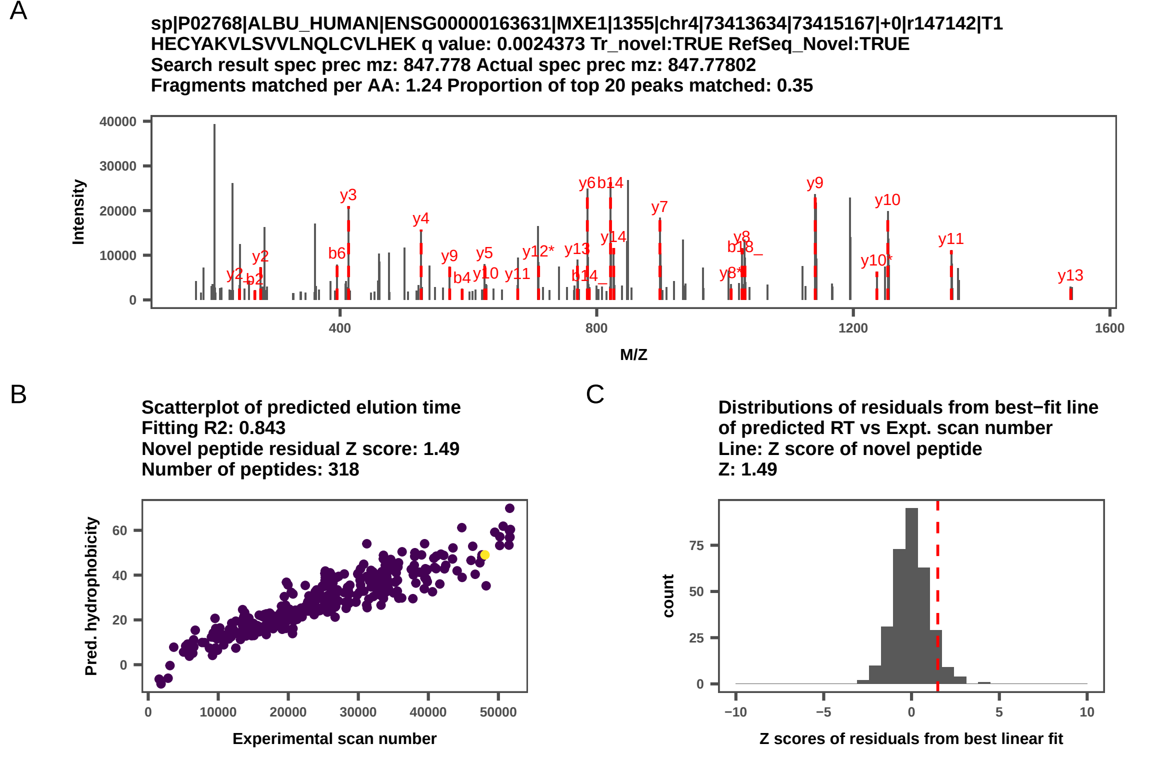
A
sp|P02768|ALBU_HUMAN|ENSG00000163631|MXE1|1355|chr4|73413634|73415167|+0|r147142|T1
HECYAKVLSVVLNQLCVLHEK q value: 0.0024373 Tr_novel:TRUE RefSeq_Novel:TRUE
Search result spec prec mz: 847.778 Actual spec prec mz: 847.77802
Fragments matched per AA: 1.24 Proportion of top 20 peaks matched: 0.35
Intensity
0
10000
20000
30000
40000
400
800
1200
1600
M/Z
y2
y2_
b2
y3
b6
y4
y9
b4
y5
y10
y11
y12*
y13
b14_
y6
b14
y14
y7
y8
b18_
y8*
y9
y10
y10*
y11
y13
B
Scatterplot of predicted elution time
Fitting R2: 0.843
Novel peptide residual Z score: 1.49
Number of peptides: 318
Pred. hydrophobicity
0
20
40
60
0
10000
20000
30000
40000
50000
Experimental scan number
C
Distributions of residuals from best−fit line
of predicted RT vs Expt. scan number
Line: Z score of novel peptide
Z: 1.49
count
0
25
50
75
−10
−5
0
5
10
Z scores of residuals from best linear fit
